# Hematological and histopathological effects of swainsonine in mouse

**DOI:** 10.1186/s12917-015-0336-6

**Published:** 2015-02-03

**Authors:** Chenchen Wu, Xiaoxue Liu, Feng Ma, Baoyu Zhao

**Affiliations:** College of Animal Veterinary Medicine, Northwest A & F University, Yangling, 712100 Shaanxi People’s Republic of China

**Keywords:** Swainsonine, Locoweed, Hemosiderin deposits, Mouse

## Abstract

**Background:**

Livestock that consume locoweed exhibit multiple neurological symptoms, including dispirited behavior, staggered gait, trembling, ataxia, impaired reproductive function and cellular vacuolar degeneration of multiple tissues due to toxicity from plant-derived alkaloids such as swainsonine.

**Results:**

Swainsonine was administered to F_0_ and F_1_ mice by intraperitoneal injection before, during and after pregnancy at the following doses: 0.525 mg/kg BW(I), 0.2625 mg/kg BW(II), 0.175 mg/kg BW(III) and 0 mg/kg BW(IV). Hemosiderin deposits were observed the lamina propria of endometrium in uterus and the red pulp of spleen. Ovary corpus lutea counts in F_0_ mice were higher in swainsonine-treated mice compared to control mice. Indirect bilirubin content and reticulocyte numbers were increased in swainsonine-treated F_0_ and F_1_ generation mice compared to control group (*P* < 0.05). Lactate dehydrogenase, alkaline phosphatase, aspartate aminotransferase and alanine aminotransferase content in F_0_-I and F_0_-II mice were significantly increased compared with F_0_-IV group mice (*P* < 0.05). Red blood cells, hemoglobin and mean corpuscular hemoglobin levels were significantly decreased in F_0_ and F_1_ mice compared with the control group (*P* < 0.05).

**Conclusions:**

Swainsonine exerts effects on estrus period and reproductive ability, and offspring of dams dosed with swainsonine were affected in-utero or from nursing. Damage to liver, uterus and spleen, as well as hematological changes, are observable before neurological symptoms present.

## Background

Locoweeds are perennial herbaceous plants of the *Astragalus* spp and *Oxytropis* spp. containing the toxic indolizidine alkaloid swainsonine [1]. Locoism causes significant economic losses to the livestock industry on western grasslands in China and the United States [2]. Swainsonine, a trihydroxy indolizidine alkaloid, is the primary toxin in locoweeds [1]. *Astragalus* and *Oxytropis* species that contain swainsonine are found on multiple continents, and have poisoned animals in South America and Asia [3,4]. Early studies demonstrated that natural or experimental long-term ingestion of swainsonine-containing plants causes serious disorders in reproductive functions of livestock (cattle, sheep, horses and goat), including failure to conceive and early embryo loss or abortion, resulting in great economic losses to the livestock industry [5-8]. Therefore, various animal models have been used to study the toxic effects of swainsonine on reproduction and development, including goat, sheep and cattle. Locoweed poisoning is usually chronic, and the toxic symptoms are observed after a few weeks of locoweed feeding. Mice were fed a small quantity of locoweed for four months, demonstrated that pathological and clinical damage to internal organs and neuronal processes were reversible [9]. In this study, we then selected four groups of mice to treat with either vehicle control or swainsonine (10 each group, F_0_-I: 0.525 mg/kg BW; F_0_-II: 0.2625 mg/kg BW; F_0_-III: 0.175 mg/kg BW and F_0_-IV: 0 mg/kg BW). After treatment with swainsonine for two weeks, female mice were mated to untreated male mice, and pups were kept with dams for one month. We sacrificed dams and offspring and observed swainsonine toxicity effects on internal organs via histopathological analysis as well as altered hematological and blood biochemical parameters in both parent and offspring mice.

## Results

### TLC detection

**Figure 1:**
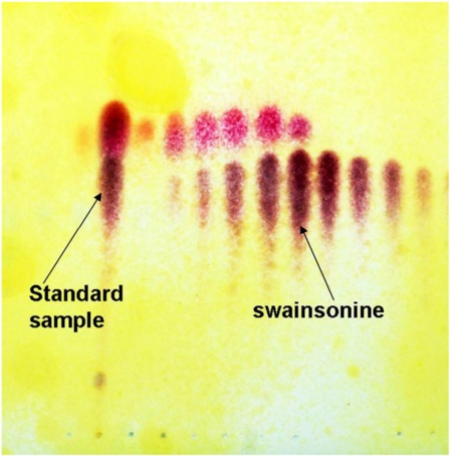
**Thin-layer chromatography of swainsonine.** The standard swainsonine sample (left arrow). The swainsonine is represented by the deep purple spots (right arrow).

All extracts were collected using column chromatography, which was placed on the thin layer plate using the capillary sample. Figure 1 show a developed TLC plate. This purple colored spots are swainsonine, the rose red colored spots are the swainsonine analogs as determined by comparison with the swainsonine standard.

### Histological effects of swainsonine treatment in F_0_ and F_1_ mice

Examination of heart, lung and kidney of treated F_0_ and F_1_ mice revealed no marked changes **(data not shown)**.

**Figure 2:**
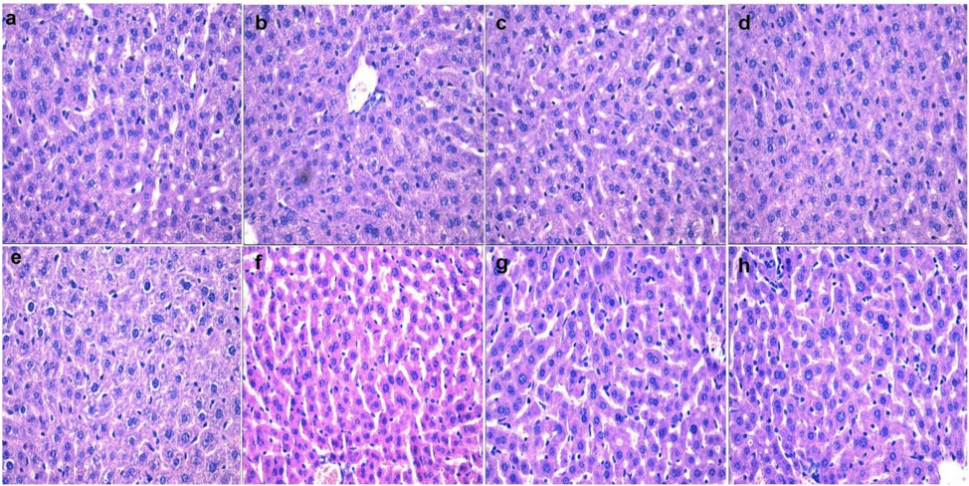
**Histological changes in F**_**0**_ **and F**_**1**_ **mice after swainsonine treatment. a-d** represent changes in the liver of F_0_-I, F_0_-II, F_0_-III and F_0_-IV(×400); **e-h** represent changes in the liver of F_1_-I, F_1_-II, F_1_-III and F_1_-IV (×400).

Histological changes in liver from swainsonine administration are shown in Figure 2 (a-d). Livers of F_0_ swainsonine-treated mice displayed few differences compared with their controls, with cellular infiltrates consisting mostly of inflammatory cells, neutrophils and granulocytes in F_0_-I, F_0_-II and F_0_-III mice. No histopathological differences were noted between F_**1**_-I, F_**1**_-II, F_**1**_-III and F_**1**_-IV control mice (Figure 2 (e-h)).

**Figure 3:**
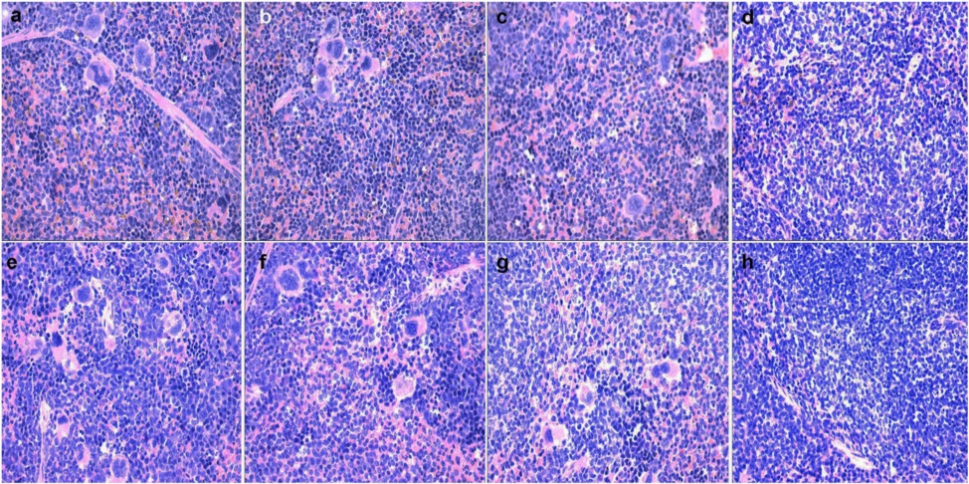
**Histological changes in F**_**0**_ **and F**_**1**_ **mice after swainsonine treatment. a-d** represent changes in the spleen of F_0_-I, F_0_-II, F_0_-III and F_0_-IV (×400); **e-h** represent changes in the spleen of F_1_-I, F_1_-II, F_1_-III and F_1_-IV (×400).

Histological analysis indicated important alterations in the spleen and uterus. As evident in Figure 3 (a-h), dose-related expansion of splenic red pulp was characterized by large numbers of inflammatory cells and lymphocytes, hypertrophy of splenic cells and a considerable number of macrophages and megakaryocytes. Increased extramedullary hemosiderin deposition were also observed in the red pulp of spleen in F_0_ and F_1_ mice (Figure 3 (a-c)). Hemosiderin deposition in the spleen of F_1_ mice was not observed (Figure 3 (e-h)).

**Figure 4:**
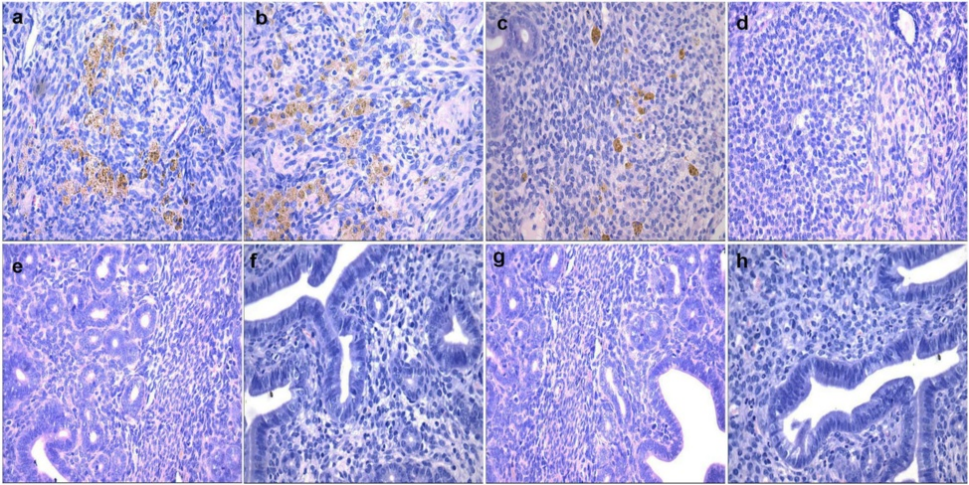
**Histological changes in F**_**0**_ **and F**_**1**_ **mice after swainsonine treatment. a-d** represent changes in the uterus of F_0_-I, F_0_-II, F_0_-III and F_0_-IV (×400); **e-h** represent changes in the uterus of F_1_-I, F_1_-II, F_1_-III and F_1_-IV (×400).

Histological alterations in uterus of mice exposed to swainsonine were more noticeable, and this effect was independent of dose (Figure 4). Hemosiderin deposits were observed in the lamina propria of endometrium in uterus of F_0_ generation mice treated with swainsonine compared with their controls (Figure 4 (a-d)). Focal collection of large numbers of neutrophils were seen in uterus mucosa of F_0_ generation mice (Figure 4 (a-c)). However, no noticeable alterations in uterus of F_1_ mice were observed (Figure 4 (e-h)).

**Figure 5:**
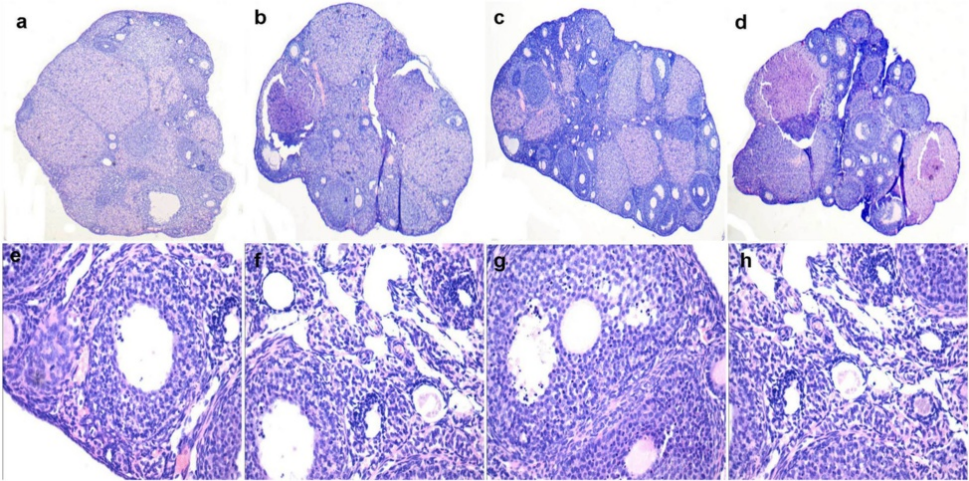
**Histological changes in F**_**0**_ **and F**_**1**_ **mice after swainsonine treatment. a-d** represent changes in the ovary of F_0_-I, F_0_-II, F_0_-III and F_0_-IV (×100); **e-h** represent changes in the ovary of F_1_-I, F_1_-II, F_1_-III and F_1_-IV (×400).

Histopathological analysis of ovaries in swainsonine-treated mice revealed dose-dependent changes compared with controls (Figure 5 (a-d)). F_0_-I and F_0_-II mice displayed decreased numbers of primordial and primary follicles compared to F_0_-IV controls. F_0_-I and F_0_-II mice exhibited increased numbers and size of corpus lutea compared with F_0_-IV control mice (Figure 5 (a-d)). However, no histopathological changes in the ovary of F_1_ mice were observed (Figure 5 (e-h)).

### Biochemical marker characterization of swainsonine-treated mice

Indirect bilirubin (IBIL) content of F_0_-I, F_0_-II and F_0_-III mice was significantly increased when compared with F_0_-IV controls (*P* < 0.05). Lactate dehydrogenase (LDH), alkaline phosphatase (ALP), aspartate aminotransferase (AST) and alanine aminotransferase (ALT) content of F_0_-I and F_0_-II mice were significantly increased compared with F_0_-IV controls (*P* < 0.05). Furthermore, indirect bilirubin (IBIL) level of F_1_-I, F_1_-II and F_1_-III mice were significantly increased compared with F_1_-IV controls (*P* < 0.05). Examination of lactate dehydrogenase (LDH), alkaline phosphatase (ALP), aspartate aminotransferase (AST) and alanine aminotransferase (ALT) content in swainsonine-treated F_1_ mice compared with controls revealed no statistically significant differences (Table 1).

**Table 1:** **Serum marker assessment in swainsonine-treated mice**

	**LDH** **U/L**	**ALP** **U/L**	**AST** **U/L**	**ALT** **U/L**	**IBIL** **mg/dL**
**F0-I**	783.9 ± 96.2*	269.84 ± 31.58*	184.32 ± 12.5*	65.87 ± 8.57*	0.43 ± 0.03*
**F0-II**	718.5 ± 95.7*	221.47 ± 32.85*	178.7 ± 10.8*	59.78 ± 9.55*	0.37 ± 0.05*
**F0-III**	623.8 ± 99.3	193.58 ± 35.87	120.87 ± 11.8	48.47 ± 8.77	0.029 ± 0.02*
**F0-IV**	587.2 ± 95.8	188.97 ± 27.31	98.11 ± 9.36	41.05 ± 9.58	0.11 ± 0.011
**F1-I**	547.54 ± 93.25	214.85 ± 7.5	116.58 ± 9.32	47.32 ± 8.50	0.28 ± 0.03*
**F1-II**	551.87 ± 90.58	218.55 ± 8.1	111.56 ± 9.65	45.36 ± 8.01	0.20 ± 0.01*
**F1-III**	569.65 ± 85.79	203.51 ± 7.5	105.8 ± 12.21	42.11 ± 7.59	0.18 ± 0.02*
**F1-IV**	554.88 ± 80.69	198.65 ± 6.8	92.34 ± 8.65	38.25 ± 7.20	0.095 ± 0.02

The values are the mean ± S.D.

*Significantly different from the control group at same generation (*P* < 0.05).

### Hematological characterization of swainsonine-treated mice

Examination of F_**0**_ dams revealed that WBCs in F_0_-I, F_0_-II and F_0_-III treatment groups were not significantly different from F_0_-IV controls (*P* > 0.05). RBCs, and levels of Hb, HCT, PLT, and MCH in F_0_-I, F_0_-II and F_0_-III treatment groups were significantly decreased compared with F_0_-IV controls (*P* < 0.05). MCV and reticulocyte levels in F_0_-I, F_0_-II and F_0_-III treatment groups were significantly increased compared with F_0_-IV controls (*P* < 0.05). Examination of F_1_ mice revealed that reticulocytes in F_1_-I, F_1_-II and F_1_-III treatment groups were significantly increased compared with F_1_-IV controls (*p* < 0.05). WBCs counts in F_1_-I, F_1_-II and F_1_-III treatment groups were not significantly different from F_1_-IV controls (*P* > 0.05). RBCs, Hb and MCH levels in F_1_-I, F_1_-II and F_1_-III treatment groups were significantly decreased compared with F_1_-IV controls (*P* < 0.05). HCT levels in F_1_-I group mice were significantly decreased compared with F_1_-IV control mice (*P* < 0.05), and MCV levels in F_1_-I and F_1_-II mice were significantly increased compared with F_1_-IV control mice (*P* < 0.05) (Table 2).

**Table 2:** **Hematological assessment in swainsonine-treated mice**

	**WBC × 10** ^**9**^ **/L**	**RBC × 10** ^**12**^ **/L**	**Hb g/L**	**HCT %**	**MCV fL**	**PLT × 10** ^**9**^ **/L**	**MCH fl**	**Reticulocytes %**
**F0-I**	7.98 ± 1.12	6.01 ± 1.54*	100.58 ± 21.58*	0.36 ± 0.03*	78.32 ± 6.58*	519.74 ± 53.9*	39.95 ± 7.58*	5.54 ± 0.78*
**F0-II**	7.21 ± 1.08	6.37 ± 1.23*	108.58 ± 25.46*	0.40 ± 0.03*	76.32 ± 6.68*	523.8 ± 48.5*	40.01 ± 6.52*	4.85 ± 0.85*
**F0-III**	7.19 ± 1.10	6.58 ± 1.15*	112.87 ± 26.54*	0.41 ± 0.02*	72.58 ± 6.98*	548.9 ± 51.25*	42.11 ± 7.56*	4.56 ± 0.96*
**F0-IV**	7.85 ± 1.75	8.45 ± 1.12	153.77 ± 20.58	0.53 ± 0.05	54.25 ± 5.44	624.88 ± 58.5	52.10 ± 7.01	2.13 ± 0.58
**F1-I**	7.89 ± 0.95	6.58 ± 1.12*	121.69 ± 25.41*	0.51 ± 0.025*	69.58 ± 6.32*	588.39 ± 56.21*	40.88 ± 7.85*	4.38 ± 0.29*
**F1-II**	7.01 ± 0.65	7.05 ± 1.25*	125.6 ± 23.15*	0.59 ± 0.035	65.32 ± 7.32*	605.81 ± 63.5*	42.02 ± 7.96*	4.18 ± 0.74*
**F1-III**	7.95 ± 0.85	7.55 ± 1.30*	139.85 ± 32.15*	0.60 ± 0.04	60.25 ± 5.91	632.87 ± 65.21	44.32 ± 8.81*	3.66 ± 0.95*
**F1-IV**	7.75 ± 1.23	8.36 ± 1.05	149.85 ± 23.56	0.62 ± 0.04	58.65 ± 5.64	658.2 ± 63.9	53.53 ± 8.21	2.07 ± 0.66

The values are the mean ± S.D.

*Significantly different from the control group at same generation (*P* < 0.05).

## Discussion

Swainsonine, a trihydroxy indolizidine alkaloid, is the main toxin in locoweed. The structure of the swainsonine cation is similar to the structure of mannose, and it has a higher affinity than mannose for mannosidase [10]. Swainsonine is a well-known inhibitor of lysosomal ɑ-mannosidase and Golgi ɑ-mannosidase II. Swainsonine induces toxicity through inhibition of ɑ-mannosidase and subsequent glycoprotein synthesis. This enzymatic dysfunction causes accumulation of complex oligosaccharides in lysosomes as well as the production of a mixture of mannose and asparagine polysaccharides, resulting in vacuolar degeneration in multiple cells [11]. Clinical symptoms in livestock are characterized by neurological and behavioral disorders, gait abnormalities, difficulty standing, abnormal posture, emaciation, reproductive disorders and cellular vacuolar degeneration of multiple tissues by pathological observation [12,13]. However, we observed two generations of mice and show organ selective vacuolar degeneration by mice given swainsonine via pathological observation. We found hemosiderin deposition in spleen and enlargement of spleen in F_0_ and F_1_ mice, and a large amount of hemosiderin deposition in uterus in F_0_ mice. When animals are fed a dose of swainsonine arrive to a certain time, the vacuolar degeneration of pathological change will show in the internal organs [9]. Therefore, we think that this experiment period did not arrive to a certain time that vacuolar degeneration found in organ. However, in our previous experiment, we also found hemosiderin deposition in spleen of rat and goat using a different dose of swainsonine [14]. We posited that some tissue bleeding occurred after swainsonine administration and found that hemosiderin deposition leads to damage in some tissues. Whether the presence of hemosiderin deposition can be used as a pathological marker of swainsonine poisoning requires further research.

The experiment results showed that two ways were not significantly different between irrigation and intraperitoneal injection by Liu Tianya [15]. Therefore, we selected the way of intraperitoneal injection for give mice to swainsonine. In this study, we demonstrate that swainsonine exerts hepatotoxicity in F_0_ mice. Alterations in liver weight and histopathological changes in liver of swainsonine- treated mice were slight. Liver from swainsonine-treated mice showed cellular infiltrates consisting mostly of inflammatory cells and neutrophil granulocytes. Significant increase of liver weight and significant alterations in levels of AST, ALT and ALP in plasma may indicate hepatic injury in F_0_ mice given swainsonine. The elevations of ALT, AST and ALP observed in swainsonine-treated mice may, in part, be due to the hepatic hypertrophic effect of swainsonine and/or may also represent borderline chronic liver toxicity [16,17]. Increased LDH activity levels have been observed in conditions of chemical stress when high levels of energy are required in a short period of time [18]. In the present study, LDH was significantly increased in F_0_ mice. However, no significant differences in biochemical markers were found between treatment and control mice F_1_ mice. This is consistent with the lack of histopathological changes in liver of F_1_ mice.

The present study identifies important histological alterations in the spleen in F_0_ and F_1_ mice, namely expansion of red pulp with vascular congestion. Furthermore, the endometrium of the uterus displayed notable deposition of hemosiderin granules in a swainsonine-treated dose-dependent manner in F_0_ mice. The molecular weight of swainsonine is small enough to penetrate the placental barrier and expose offspring in-utero. A major function of the spleen is to remove aged and damaged erythrocytes from the blood [19]. Excess hemosiderin deposition in spleen can result in the destruction of macrophages and the release of the contents such as iron, toxic compounds and/or its metabolites into spleen [20]. Toxic effects in both F_1_ and F_0_ mice include reduction of RBCs, reduction in levels of Hb, HCT, PLT and MCH, as well as an increase in the number of reticulocytes, suggesting the development of anemia [21]. Significant increases in IBIL were observed in F_0_ and F_1_ mice given swainsonine. The increase of IBIL further indicates that swainsonine could be damaging red blood cells. When organs bleed, red blood cells are phagocytized by macrophages and degraded by lysosomes; Fe^3+^ of hemoglobin from lysed red blood cells can combine with protein to form hemosiderin. Because we observed decreased RBCs, and decreased levels of Hb, MCH and MCV as well as an increase in reticulocytes, we suspect that our dose levels of swainsonine may lead to anemia.

Swainsonine is water-soluble and rapidly distributed to many parts of the body. In previous studies, swainsonine concentrations varied widely in various tissues and organs of sheep that had ingested locoweed [22-24]. In this study, uterus of swainsonine-treated F_0_ mice was heavily damaged. This was characterized by the presence of hemosiderin deposits in the lamina propria of endometrium in uterus of F0 mice in this study. In ovary, F_0_-I and F_0_-II mice displayed decreased numbers of primordial and primary follicles compared to F_0_-IV controls. In addition, F_0_-I and F_0_-II mice displayed increased size and number of corpus lutea compared to F_0_-III and F_0_-IV. The lesions in ovary and uterus were dose-dependently observed in F_0_-I, F_0_-II and F_0_-III treatment groups. However, F_1_ did not display notable histopathological changes in the uterus and ovary. Swainsonine easily accumulates in uterus at high concentrations, which may impair uterus and ovary function and cause toxicity. In the present study, the uterus suffered noticeable damage, which led to a decline in the rate of conception, an increase in the rate of abortion and increases in stillborn births. It is suspected that significant early embryonic loss occurs in cattle and sheep grazing locoweed, and there are documented effects of swainsonine on oocyte maturation, fertilization and subsequent embryonic implantation and development [24]. Increased numbers of corpus lutea in ovary can lead to delayed or halted estrus. The pathological lesions we observed, combined with altered hematological and serum biochemical parameters in swainsonine-treated mice, suggest that exposure to swainsonine may lead to inhibition of reproductive performance under certain doses.

## Conclusions

Based on sub-chronic toxicity results, our data establishes effects of swainsonine on reproductive toxicity in a mouse model. In addition, we found that swainsonine can cause hematological changes and lesions in spleen, uterus, ovary and liver. Furthermore, we provide evidence of trans-generational swainsonine toxicity through placental barrier and milk. Spleen, heart, liver, lung, kidney, uterus and ovary were among the organs affected in offspring of dams given swainsonine. Large amounts of hemosiderin deposition in uterus and spleen were observed in the parent generation. We present evidence that hemosiderin deposition may preclude vacuolar degeneration in some tissues of mice given swainsonine. Alterations in hematological and histopathological parameters suggest a link to anemia and decreases in reproduction ability. Our data suggest that anemia and organ-specific hemosiderin deposition followed by destruction of red blood cells are clinical features of swainsonine-treated mice. However, further research is needed to elucidate specific mechanisms of swainsonine toxicity.

## Methods

### Ethical statement

Female *Rattus norvegicus* mice were supplied by the Animal Center of the Fourth Military Medical University. During the experiment, mice were housed individually in polypropylene cages with laboratory grade pine shavings as bedding. Mice were maintained in a controlled environment with temperature maintained between 19-25°C, relative humidity maintained between 40-70%, >8 air changes/hour, and with a 12:12-h light: dark cycle. The experimental procedures were in accordance with the Ethical Principles (Animal [Scientific Procedures] Act 2012) in Animal Research adopted by the China College of Animal Experimentation and were approved by the College of Veterinary Medicine- Northwest A&F University.

### Study design

#### Extraction of swainsonine from locoweed

The aerial portion of *Oxytropis kansuensis* was collected from the grassland in Tianzhu city, Gansu province in July 2011. The plants were then taxonomically identified by Zhao Bao-Yu, College of Veterinary Medicine, Northwest A and F University, China. The plants were subsequently dried in the shade, finely ground and comminuted.

The extraction and analysis method of swainsonine from *Oxytropis kansuensis* was conducted as previously described [25].

### Analysis of swainsonine

Thin-layer chromatography (TLC) detection was performed on silica gel G precoated plates with the developing solvents chloroform:methanol:ammonia:water (70:26:2:2, V/V), chloroform:methanol:ammonia:water (70:26:10:10, V/V), and methanol: ethylacetate: ammonia (4:1:1,V/V) and modified potassium heptaiodobismuthate reagent or H_2_O_2_/10% acetic anhydride in EtOH/Ehrlich’s reagent was the chromogenic agent.

The extracts were dissolved in methanol, spotted onto the GF254 silica gel G precoated plates. The plates were developed with an ascendant run after saturation with the mobile phase in a s glass chamber for 5–10 min. The plates were dried when the mobile phase was 10 mm from the front edge of the plates. The plates were stained successively with a spray of H_2_O_2_ (heated for 10 min in an oven at 115°C), a spray of 10% acetic anhydride in dehydrated alcohol (heated at the same temperature until the smell of acetic anhydride disappeared) and finally a spray of Ehrilich’s reagent (heated for 15 min at 120°C). The color of the spots in each plate was recorded, and the R_f_ was determined [25].

### Animals to experimental groups

Female mice (N = 40, six weeks old) were divided into four equal groups of 10 mice (10 each group, F_0_-I: 0.525 mg/kg BW; F_0_-II: 0.2625 mg/kg BW; F_0_-III: 0.175 mg/kg BW and F_0_-IV: 0 mg/kg BW). All mice were administered swainsonine by intraperitoneal injection 14 days before the mating period followed by re-administration every three days. After this pre-mating period, the treated mice were transferred to the home cage of a male in the same group and cohabited on a 1:1 basis until achievement of successful mating. During the mating period, mice were examined daily for presence of vaginal plugs, and a vaginal plug was considered evidence of successful mating. Pregnant dams continued to receive swainsonine every three days via intraperitoneal injection throughout parturition and the lactation. Upon weaning of four-week-old pups (F_1_), the dams (F_0_) were sacrificed, and the liver, kidney, heart, spleen, lung, uterus and ovary were collected. In total, F_0_ mice were given swainsonine for six to eight weeks in the whole experiment.

Female offspring (F_1_) of treated dams were selected from each of the four treatment groups (40 F_1_ mice in total, 10 from each F_0_ treatment group). The F_1_ offspring were not treated with swainsonine, however, the dams continued to be dosed while nursing their F_1_ pups. The F_1_ offspring were then sacrificed after approximately 1 month of nursing. The liver, kidney, heart, spleen, lung, uterus, and ovary were collected.

All F_0_ group mice received intraperitoneal injections of swainsonine once every three days under aseptic conditions. Upon sacrifice, the liver, kidney, heart, spleen, lung, uterus and ovary were trimmed of extraneous fat and weighed immediately.

### Histopathological preparation

All tissues were removed and fixed in 10% formaldehyde at room temperature. The tissue samples were then dehydrated and embedded in paraffin according to standard histological procedures. Serial cross-sections of 3 μm were prepared from each organ. The sections were mounted and stained with hematoxylin-eosin.

### Hematological assessment

White blood cells (WBCs), red blood corpuscles (RBCs), hemoglobin (Hb), hematocrit (HCT), mean corpuscular volume (MCV), blood platelets (PLTs), mean corpuscular hemoglobin (MCH) and reticulocyte counts were determined by automatic hematological analyzer, MEK-8222 K (TOA Medical Electronics, Kobe, Japan).

### Blood biochemical analysis

Blood was collected when mice were sacrificed. Lactate dehydrogenase (LDH), aspartate aminotransferase (AST), alanine aminotransferase (ALT), alkaline phosphatase (ALP) and indirect bilirubin (IBIL) were quantitated using the Beckman Synchron CX7 Delta Chemistry Analyzer (Beckman, USA).

### Statistical methods

The statistical software “Statistical Product and Service Solutions” (SPSS V11.3) was used to determine statistically significant differences between treatment groups and the control group. A one-way ANOVA was used to evaluate the homogeneity of the data, and a least squared differences model or Dunnett’s multiple comparison test were then used. Values of *p* < 0.05 were considered significant. The data are presented as the group mean values ± SD (standard deviation).
